# Bean Syndrome in a Child Treated with Sirolimus: About a Case

**DOI:** 10.1155/2022/8245139

**Published:** 2022-05-24

**Authors:** Ayad Ghanam, Aziza Elouali, Merouane Nour, Maria Rkain, Noufissa Benajiba, Abdeladim Babakhouya

**Affiliations:** ^1^Department of Pediatrics, Mohammed VI University Hospital, Oujda, Morocco; ^2^Faculty of Medicine and Pharmacy, Mohammed Ist University, Oujda, Morocco; ^3^Department of Pediatric Surgery, Mohammed VI University Hospital, Mohammed I University, Oujda, Morocco

## Abstract

Bean syndrome (BS) or blue rubber bleb nevus syndrome is a rare clinical entity characterized by venous malformations mainly in the skin and digestive tract, whose hemorrhagic complications can be life threatening. We report a case of Bean syndrome in a 3-year-old child of nonconsanguineous parents, in whom the diagnosis of miliary hemangiomatosis was initially made in view of a huge mass on the left thigh, taking the knee, and then the progressive appearance of a skin disorder with bluish swellings of variable sizes spread over the whole body. The patient was put on beta-blockers but without improvement. The evolution was marked by an increase in the volume of the thigh mass. Ultrasound exploration coupled with Doppler imaging revealed the presence of angiomas in the thigh, requiring emergency surgery following a large hemorrhage. The patient underwent sclerotherapy. At the age of 18 months, the child returned with severe anemia and melena. The abdominal CT scan showed gallbladder intussusception secondary to an angioma requiring intestinal resection for hemostasis. At the age of three years, the angiomas worsened with an increase in volume, particularly on the face. The association of the cutaneous and digestive involvement of these venous malformations made us rectify the diagnosis. The patient was put on sirolimus (rapamycin), 2 mg/m^2^, with good evolution with a delay of 18 months; the patient presents no more episodes of bleeding with regression of the size of cutaneous angiomas. This observation underlines that BS is difficult to diagnose because of its low frequency, that sirolimus was effective and well tolerated in our patient, and that it can be suggested as a good and safe therapeutic option.

## 1. Introduction

Bean syndrome or blue rubber bleb nevus syndrome is a rare disease characterized by venous malformations mainly in the skin and digestive tract, with life-threatening hemorrhagic complications. We report a case in a child successfully treated with sirolimus.

### 1.1. Observation

**Figure 1:**
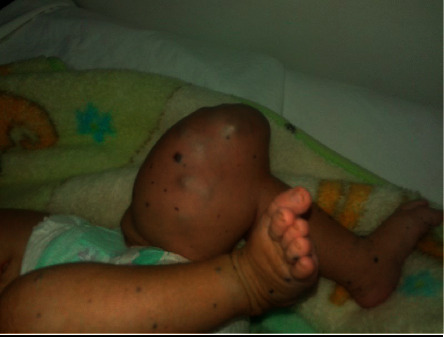
Huge cutaneous angioma of the left thigh taking the left knee at the age of 6 months.

**Figure 2:**
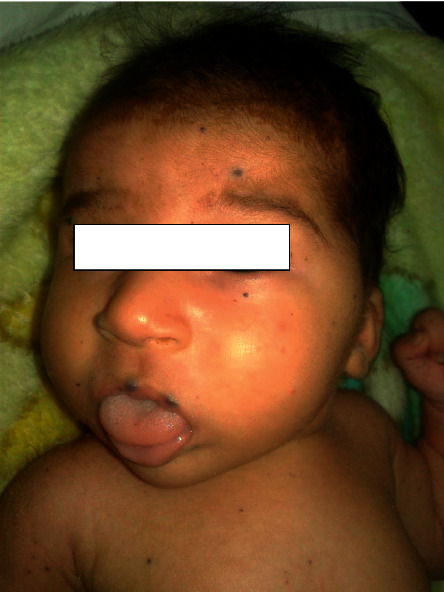
Diffuse cutaneous angiomas on the whole body.

**Figure 3:**
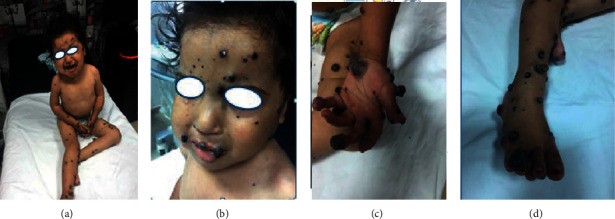
Diffuse cutaneous angiomas on the whole body (a) and cutaneous angiomas on the face (b), on the upper limbs (c), and on the lower limbs (d).

Child aged 3 years, from a pregnancy estimated at term, of nonconsanguineous parents, followed since birth for a malformative venous syndrome with a huge mass on the left thigh, taking the knee (Figure 1), and then progressive appearance of a skin disorder with bluish swellings of variable sizes (0.5–3 cm) spread all over the body (face, thorax, abdomen, and upper and lower limbs) (Figure 2) . The patient was put on propranolol at a dose of 3 mg/kg/day, administered in 2 doses but without improvement. The evolution was marked by an increase in the volume of the thigh mass. Ultrasound exploration coupled with Doppler showed the presence of angiomas in the thigh requiring an emergency surgical intervention following a large hemorrhage. The patient received blood transfusion with sclerotherapy. At the age of 18 months, the child returned with severe anemia with melena requiring an emergency transfusion, complicated by the appearance of heavy rectal bleeding. An abdominal CT scan showed a gallbladder intussusception secondary to an angioma requiring an intestinal resection for hemostasis. The postoperative course was simple. Thereafter, we noted the reappearance of melenas with the need for frequent transfusions. At the age of three years, the angiomas worsened with an increase in volume, diffuse cutaneous angiomas on the whole body (Figure 3(a)), face (Figure 3(b)), upper limbs (Figure 3(c)), and lower limbs (Figure 3(d)). The association of the cutaneous and digestive involvement of these venous malformations made us rectify the diagnosis. The patient was put on sirolimus (rapamycin): 2 mg/m^2^, with good evolution. The child has been followed regularly since then in consultation with a delay of 18 months, and the patient no longer presents episodes of bleeding with regression of the size of cutaneous angiomas.

## 2. Discussion

Bean syndrome or “blue rubber bleb naevus” or diffuse angiomatosis was first reported in 1860, in a patient who presented with asphyxia following acute hemorrhage from a parotid tumor. He also had multiple angiomas in the skin and digestive tract. Named after William Bennet Bean, it is this author who in 1958 described the skin lesions that are compressible to palpation as a “rubber teat” [1]. It is a rare clinical entity characterized by venous malformations mainly in the skin and digestive tract, whose hemorrhagic complications can be life threatening [2]. These venous malformations are multifocal, most often affecting the skin and the digestive tract, and can also be located in the brain, lungs, kidneys, bones, eyes, and even other organs. The lesions are usually present in childhood as in our patient's case but can also develop later life [3]. The prevalence remains unknown but more than 200 cases have been described. It is a disseminated and sporadic venous malformation that could be familial with an autosomal dominant transmission secondary to a mutation on chromosome 9p [2]. The main clinical signs result from acute or chronic bleeding from multiple lesions of the digestive tract, which are mainly located in the small intestine. Acute bleeding presents as hematemesis, melena, or rectal bleeding. The lesions are cutaneous and visceral, in particular gastrointestinal damage responsible for haemorrhages, urinary, hepatic, splenic, cerebral, and pulmonary damage [4]. The extra-digestive lesions can be responsible for orthopedic deformity [5] as in the case of our patient where the mass caused deformity of the left knee, pulmonary embolism, pulmonary hypertension, hemothorax, hemopericardium [6], exophthalmia [7], thrombocytopenia, and chronic consumption coagulopathy. Skin lesions may be macular, papular, nodular, or pedunculated, and reddish or purplish. They are multiple and most often nonbleeding [8]. They can be classified into 3 types [9]. Type 1 is a large deforming lesion that may obstruct the adjacent organ, type 2 is the most frequent type, the lesion is blue and crumpled like a “rubber teat” covered with skin [10], and type 3 is a macular or papular lesion with blue-black color that may blanch on pressure. These skin lesions may be present at birth as in our patient's case or may appear over the years [9]. Cutaneous angiomas can become painful; this pain is due to the tension of these lesions in certain positions and also to localized thrombosis, at the origin of phleboliths which are round calcifications pathognomonic of venous malformations and clearly visible on radiographs [9]. Digestive hemorrhage is the most frequent complication of intestinal involvement in Bean syndrome, but intestinal intussusception is also described in a few publications [11], as was the case for our patient. Diagnostic methods include blood count, endoscopy, ultrasound, CT scan, magnetic resonance imaging, and histology. In the absence of appropriate treatment, affected patients suffer from anemia due to chronic bleeding that requires iron supplementation and repeated transfusions. Malignant transformation of the lesions has not been described so far. Pharmacological treatment with octreotide, corticosteroids, and hormone therapy have been described, but none of them has demonstrated adequate control of anemia. The endoscopic approach to remove accessible lesions or surgical resections is proposed, but the high number of malformations along the small intestine prevents complete resection of lesions. Successful treatment with sirolimus has been reported in several vascular and lymphatic malformations. The first use of sirolimus in the management of SB was in 2012, and since then, a few more cases have been reported [12]. In pediatric patients, the dose is calculated according to the body surface area at 0.5–2 mg/m^2^ [13]. The most common adverse effects are hyperlipidemia (51%), thrombocytopenia (10–18%), and leukopenia (10–18%) [13]. Other adverse effects are an increased risk of mucosal infections, arthralgias, and alterations in liver and kidney functions. The prognosis of BS depends on the main hemorrhagic complications and the location of these angiomas in relation to vital organs.

## 3. Conclusion

The particularity of our observation lies in the rarity of Bean syndrome and confirms the effectiveness of sirolimus treatment with a good tolerance profile and could be proposed in first line in multivisceral forms.

## Data Availability

No data were used to support this study.
